# The Conceptualization, Experience, and Recognition of Emotion in Autism: Differences in the Psychological Mechanisms Involved in Autistic and Non‐Autistic Emotion Recognition

**DOI:** 10.1002/aur.70162

**Published:** 2026-01-25

**Authors:** Connor Tom Keating, Carmen Kraaijkamp, Jennifer Louise Cook

**Affiliations:** ^1^ School of Psychology University of Birmingham Birmingham UK; ^2^ Department of Experimental Psychology University of Oxford Oxford UK; ^3^ Faculty of Psychology and Neuroscience Maastricht University Maastricht the Netherlands

**Keywords:** autism, concepts, emotion, facial expressions, social cognition

## Abstract

Existing literature suggests that differences between autistic and non‐autistic people in emotion recognition might be related to differences in how these groups experience emotions themselves. Specifically, autistic individuals may show differences in the consistency of emotional experiences, the ability to distinguish between emotions, and/or their semantic understanding of emotions. In this study, we empirically tested this claim by (1) investigating whether autistic and non‐autistic adults differed in the consistency and/or differentiation of their emotional experiences, and their understanding and differentiation of emotion concepts after controlling for alexithymia, and (2) assessing the contribution of these emotional abilities to emotion recognition. To this end, a total of 58 autistic and 59 non‐autistic individuals, matched on age, sex, and non‐verbal reasoning ability, completed a series of validated questionnaires and computer‐based emotion tasks. We found no group differences in emotional consistency, emotion differentiation, and understanding or differentiation of emotion concepts after controlling for alexithymia. For non‐autistic people, the ability to differentiate one's own emotions contributed to enhanced emotion recognition. Although having more differentiated emotion concepts (indirectly) contributed to elevated emotion recognition for non‐autistic people, having a more precise understanding of emotion concepts contributed to emotion recognition for autistic people. Our findings demonstrate that there are differences in the psychological mechanisms involved in autistic and non‐autistic emotion recognition. The results of the current study pave the way for future systems to help *both* autistic and non‐autistic people to more accurately recognize emotional facial expressions.

## Summary

This study investigated how autistic and non‐autistic adults experience and understand emotions, and how these abilities relate to recognizing emotions in others. We found that autistic and non‐autistic adults did not differ in (1) how consistently they experienced emotions, (2) their understanding of emotion terms, and (3) their ability to distinguish between different emotional experiences or emotion terms. For non‐autistic individuals, recognizing emotions was linked to how well they could tell apart emotion terms and their own feelings. For autistic individuals, a clear understanding of emotion terms was associated with better emotion recognition. These insights could support the development of tools to enhance emotion recognition for both groups.

## 1 Introduction

Autism is a neurodevelopmental condition, characterized by socio‐communicative differences and repetitive patterns of behaviors, interests or activities (American Psychiatric Association 2013). Although not considered a diagnostic feature, emotion recognition has been a topic of interest in autism research for over three decades because it is thought that difficulties in this area may contribute to social differences (Baron‐Cohen et al. 2009). To date, the majority of emotion recognition research has aimed to determine whether differences exist between autistic (using identity‐first language, in accordance with the majority preference of the autistic community (Keating, Hickman, et al. 2023; Geelhand et al. 2023)) and non‐autistic individuals (Keating and Cook 2020; Harms et al. 2010; Lozier et al. 2014; Yeung 2022). This literature is famously mixed (see Keating and Cook 2020; Yeung 2022): Some studies show differences in emotion recognition between groups, while others find no differences, or emotion‐specific difficulties (e.g., in recognizing angry expressions (Lozier et al. 2014; Ashwin et al. 2006; Bal et al. 2010; Brewer et al. 2016; Keating, Fraser, et al. 2022; Leung et al. 2019; Song and Hakoda 2018)). Here, instead of focusing on assessing whether there are group differences in emotion recognition, we explore whether there are differences in the *way in which* autistic people read emotional expressions. That is, we ask whether autistic and non‐autistic people typically employ different *mechanisms* to recognize the emotions of others.

One way in which autistic and non‐autistic people may differ in emotion recognition concerns the extent to which they draw on their own emotional experiences when interpreting others' emotions. A person's internal emotional landscape is an important contributor to how well they can recognize the emotions of others (see Keating and Cook 2023b). For instance, individuals who have more *consistent* and *differentiated* emotional experiences typically find it easier to successfully recognize other people's emotions. Our previous work provided empirical support for this in a large (*N* = 193) sample of non‐autistic participants. Participants completed a two‐part “EmoMap” paradigm wherein they first viewed pairs of images each known to selectively induce either anger, happiness, or sadness (Riegel et al. 2016), and rated how similar the evoked emotions felt. They subsequently selected the image that made them feel the most angry, happy, or sad. Emotion differentiation was calculated using a multidimensional scaling algorithm to transform similarity scores into “distances” between emotions. Emotional consistency was calculated based on the logical consistency of participants' responses: If a participant selected Image A over Image B, and Image B over Image C, but then selected Image C over Image A, this would comprise an inconsistent decision and would indicate inconsistency in their emotional experience. Thus, individuals with highly consistent and differentiated emotions are consistent in their emotional responses to the images and feel very different inside when they experience anger, happiness, and sadness. Previously, we found that non‐autistic participants with more consistent and differentiated emotional experiences typically had greater emotion recognition accuracy on an independent test (Keating and Cook 2023b). At present, it is not known whether the same is true for autistic individuals.

Another potential contributing factor to emotion recognition concerns how well individuals understand semantic emotion concepts (i.e., the semantic meaning associated with the emotion) and are able to differentiate these from one another (e.g., differentiating the concept of sadness from disappointment). Contemporary theories of emotion and emerging evidence suggest that semantic emotion concepts shape how individuals “construct” both emotional experiences (i.e., inferences about how oneself is feeling) and emotion perceptions (i.e., inferences about how others are feeling) (Barrett 2006; Nook et al. 2015, 2017; Lindquist and Barrett 2008; Lindquist et al. 2015; Widen et al. 2015; Keating 2024). Specifically, these theories suggest that from childhood through adulthood, emotion concepts evolve from a “positive vs. negative” dichotomy into more differentiated multidimensional representations, producing concomitant shifts in the experience and perception of emotion (Nook et al. 2017). That is, possessing emotion concepts that are differentiated across more dimensions will encourage individuals to differentiate between their own affective experiences and others' emotional facial expressions across more dimensions (e.g., arousal and context in addition to valence). Hence, as we develop, we move away from conceptualizing, experiencing, and perceiving emotions as “good” and “bad” to conceptualizing, experiencing, and perceiving them more precisely (e.g., based on arousal, context, etc.).

Although theories to date are highly informative, they have not yet specified whether emotion concepts influence experiences and perceptions independently and directly, or whether there are indirect effects amongst these variables (one variable influences another, which influences a third variable). It could be, for example, that having precise and distinct semantic emotion concepts helps an individual to differentiate between their own emotional states, which in turn helps them to tell apart others' emotional expressions. To determine the mechanistic pathways amongst these variables, studies employing causal manipulation are necessary. However, at present, the putative direction of causality is unknown, thus making it impossible to determine which factor should be the target for manipulation. Here, research employing mediation analyses offers a potential solution, identifying the most mathematically plausible pathways, and thus opening avenues to future studies formally testing the degree of causality and directionality between these variables.

Preliminary work suggests that there may be differences between autistic and non‐autistic people in their ability to differentiate experiences and semantic concepts of emotion. Erbas et al. (2013), for example, have argued that autistic adolescents have less differentiated experiences and concepts of emotion than their non‐autistic counterparts. In support of this, these authors found that the autistic participants sorted emotion terms into fewer conceptual groupings, suggesting these individuals make less fine‐grained distinctions between emotion concepts. Autistic adolescents also had less differentiated emotional responses to emotion‐inducing images (Erbas et al. 2013). Importantly, however, this study did not control for alexithymia—a subclinical condition, highly prevalent in autistic people (Kinnaird et al. 2019), characterized by difficulties identifying and describing one's own emotions (Nemiah et al. 1976). This could be problematic as it is thought that autistic individuals' challenges with emotion processing (including emotion differentiation) may be underpinned by alexithymia, and not autism (Bird and Cook 2013). Further research is necessary to understand whether autistic people have less differentiated experiences and concepts of emotion after controlling for alexithymia.

Although research has demonstrated a role for both emotion differentiation *and* emotional consistency in the recognition of emotion (Keating and Cook 2023b), studies have not yet examined emotional consistency in the context of autism. However, it could be that emotional consistency is lower in autism (in addition to emotion differentiation as described above), thus contributing to emotion recognition difficulties. Alternatively, given that different traits and processes appear to be involved in autistic and non‐autistic emotion recognition (Brewer et al. 2016; Rump et al. 2009; Keating et al. 2023; Keating, Sowden, et al. 2026), this factor may not contribute to emotion recognition for autistic individuals at all.

In sum, it is unclear whether there are differences between autistic and non‐autistic individuals in their ability to differentiate experiences and semantic concepts of emotion, and/or the consistency of their emotional experiences, after controlling for alexithymia. Such differences could conceivably feed into challenges with recognizing others' emotional expressions. As such, the current study had two primary aims: (1) to investigate whether autistic and non‐autistic adults differed in the consistency and/or differentiation of their experiences and semantic conceptions of emotion, and (2) to investigate the contribution of these factors to emotion recognition for both autistic and non‐autistic people. In addition, to identify putative mechanistic pathways, we conducted exploratory post hoc analyses to identify whether the ability to differentiate one's own emotions mediates the relationship between the differentiation of emotion concepts and emotion recognition. Importantly, throughout, we control for alexithymia to ensure that any differences between the groups arise due to autism, and not alexithymia, as has been found in previous work (Bird and Cook 2013; Cook et al. 2013; Milosavljevic et al. 2016; Ola and Gullon‐Scott 2020).

## 2 Methods

This study was approved by the Science, Technology, Engineering and Mathematics (STEM) ethics committee at the University of Birmingham (ERN_16‐0281AP9D) and conducted in line with the principles of the revised Helsinki Declaration. All participants provided informed consent. This study was not pre‐registered.

### 2.1 Participants

A total of 58 autistic and 59 non‐autistic participants—matched on age, sex, and non‐verbal reasoning ability (NVR) (see Table 1)—took part in this study. The autistic participants were recruited via the Birmingham Psychology Autism Research Team (B‐PART) database and via emails to university mailing lists. All participants in the autism group had previously received a clinical diagnosis of Autism Spectrum Disorder from an independent clinician. The non‐autistic participants were recruited via the Research Participation Scheme database, emails to university mailing lists, and via Prolific. As expected, the autistic participants had significantly higher autism quotient (AQ) (Baron‐Cohen et al. 2001) scores than the non‐autistic participants [*U* = 384.5, *Z* = −7.24, *p* < 0.0001]. Participants' ethnicities and levels of education are reported in Supporting Information A and B, respectively.

**TABLE 1:** Means, standard deviations, and group differences of participant characteristics.

Variable	Non‐autistic (*n* = 59)	Autistic (*n* = 58)	Significance
Sex	33 female, 26 male	31 female, 24 male, 3 prefer not to say	*p* = 0.209
Age	32.27 (15.30)	33.26 (11.54)	*p* = 0.105
NVR	59.50% (13.54%)	61.11% (18.61%)	*p* = 0.707
AQ‐50	20.80 (8.54)	35.34 (7.80)	*p* < 0.001
TAS‐20	49.48 (14.25)	61.79 (11.60)	*p* < 0.001

*Note:* In the central columns, means are followed by standard deviation in parentheses. Age is in years. NVR = non‐verbal reasoning.

The chosen sample size was based on a priori power analyses conducted using G*Power (Faul et al. 2007). First, we aimed to compute the sample size needed to detect group differences in emotional consistency and emotion differentiation. We conducted two sample size calculations for an ANCOVA: one focusing on the group effect and the other focusing on the emotion × group interaction. In both calculations, we assumed a moderate effect size (Cohen's *f* = 0.30) based on the group difference in emotion differentiation (*f* = 0.33) reported by Erbas et al. (2013). As there was no prior research on emotional consistency in autism, this served as the closest available reference point (particularly as emotional consistency and emotion differentiation are related (Keating and Cook 2023b)). Across both calculations, alpha was 0.05, power was 0.8, and the number of covariates was nine (age, sex, non‐verbal reasoning, years of education, alexithymia, emotional vocabulary score, within valence conceptual distance, between valence conceptual distance, and mean definition word count). In our first calculation, which focused on the group effect, the number of groups was two (autistic, non‐autistic), and the numerator degrees of freedom was one (2 − 1). In our second calculation, which focused on the emotion × group interaction, the number of groups was six (three emotion × two groups) and the numerator degrees of freedom was two ((3 − 1) × (2 − 1) = 2). Through these calculations, we identified that the total number of participants required to detect the group and emotion × group effects at *p* < 0.05 was 90 (45 in each group) and 111 (55 and 56 per group), respectively.

Next, we aimed to compute the sample size needed to detect group differences in the understanding and differentiation of semantic emotion concepts. Given that there would be no emotion × group interaction in these statistical models, only one sample size calculation was needed for the group effect. As above, we assumed a moderate effect size (Cohen's *f* = 0.30). Alpha was 0.05, power was 0.8, the numerator degrees of freedom was one (2 − 1), the number of groups was two (autistic, non‐autistic), and the number of covariates was six (age, sex, non‐verbal reasoning, years of education, alexithymia, mean definition word count). Through this calculation, we identified that the total number of participants to detect the group effect at *p* < 0.05 was 90 (45 in each group).

Finally, we aimed to compute the sample size needed to detect the contribution of emotion differentiation to emotion recognition, reported previously (Keating and Cook 2023b). In this sample size calculation, we assumed a moderate effect size (Cohen's *f* ^2^ = 0.15), based on previous literature (Cohen's *f* ^2^ = 0.159 in Keating and Cook 2023b). Alpha was 0.05, power was 0.8, the number of tested predictors was one, and the total number of predictors was 10 (since we were confident our data‐driven models predicting emotion recognition accuracy would have no more than 10 predictors). In this analysis, we identified that 55 participants were required in each group to detect the contribution of a variable to emotion recognition at *p* < 0.05. With this sample size, we should be able to detect a moderate contribution of any variable (e.g., emotional consistency, understanding of semantic emotion concepts) to emotion recognition.

### 2.2 Community Involvement

In line with participatory research guidelines (Fletcher‐Watson et al. 2019; Keating 2021), we sought input from five members of the autism community—including autistic individuals and family members of autistic people—via the Birmingham Psychology Autism Research Team Consultancy Committee prior to conducting the study. To gain their feedback, we presented the study proposal to community members in an online meeting, summarizing the background literature, rationale, and suggested methods. We then discussed the draft task instructions and completed a few trials of each task together. To help promote openness and reduce power imbalances between researchers and community members, we (1) emphasized the importance of lived experience in enhancing the quality and relevance of research, (2) made it clear that their contributions were genuinely valued, (3) clearly communicated our openness to all feedback (stating that there were no “silly” questions or feedback), and (4) created an inclusive online environment that enabled participants to contribute either verbally or through the chat function.

The community members provided valuable feedback on various aspects of the research, including task design, instructions, and potential dissemination routes. Based on their insights, we made several changes before beginning data collection. For instance, they recommended including more frequent breaks to reduce fatigue during some tasks. In response, we divided the first part of the EmoMap paradigm into three blocks and the Emotional Vocabulary Test into four blocks, each separated by breaks. The consultants also suggested modifying the wording of instructions to maximize clarity and accessibility. As a result, we changed the instructions in the Emotional Vocabulary Test from “please define this emotion word” to “what does this emotion word mean?” Moreover, to encourage thoughtful and high‐quality responses, the community members proposed informing participants that their definitions might be reused in future research (with their consent). We incorporated this suggestion into the task instructions. These community members also offered further input—such as suggestions for recruitment channels—which helped shape various elements of our approach.

## 3 Procedures

Participants provided informed consent and then completed demographics questions, the AQ (Baron‐Cohen et al. 2001), and the Toronto Alexithymia Scale (Bagby et al. 1994) on Qualtrics. Following this, participants completed EmoMap (Keating and Cook 2023b), the point light face (PLF) emotion recognition task (Brewer et al. 2016; Sowden et al. 2021), the emotional vocabulary test (inspired by Nook et al. 2017) and the Matrix Reasoning Item Bank (MaRs‐IB) (Chierchia et al. 2019) on Gorilla.sc. All parts of the study were completed online.

### 3.1 Materials and Stimuli

#### 3.1.1 The AQ

The level of participants' autistic traits was assessed via the 50‐item AQ (Baron‐Cohen et al. 2001). This self‐report questionnaire is scored from 0 to 50, with higher scores representing higher levels of autistic characteristics. The AQ assesses five different domains: attention switching, attention to detail, communication, social skills, and imagination. The AQ is a popular tool for assessing autistic traits in both the general population and in autistic individuals (Ruzich et al. 2015; Whitehouse et al. 2011; Hickman et al. 2022; Abu‐Akel et al. 2019), and has high internal consistency (*α* ≥ 0.7) and test–retest reliability (*r* ≥ 0.8) (Stevenson and Hart 2017).

#### 3.1.2 The Toronto Alexithymia Scale

The level of participants' alexithymic traits was measured using the 20‐item Toronto Alexithymia Scale (TAS‐20) (Bagby et al. 1994). This self‐report questionnaire comprises 20 items rated on a five‐point Likert scale, ranging from 1, *strongly disagree*, to 5, *strongly agree*. Scores on this questionnaire range from 20 to 100, with higher scores indicating higher levels of alexithymic traits. The TAS‐20 is the most popular tool for assessing alexithymia and has good internal consistency (*α* ≥ 0.7) and test–retest reliability (*r* ≥ 0.7) (Bagby et al. 1994; Taylor et al. 2003).

#### 3.1.3 EmoMap

The differentiation and consistency of participants' emotional experiences was assessed using our two‐part EmoMap paradigm. In the first part, on each trial, participants viewed pairs of images from the Nencki Affective Picture System (Marchewka et al. 2014), and were required to rate how similar the emotions evoked by each of the images were. Participants made their ratings on a visual analogue scale (with a step size of 0.0001) ranging from 0, “*not at all similar*” to 10, “*very similar*.” An advantage of the EmoMap paradigm is that it allows us to measure emotion differentiation without requiring participants to translate their emotional experiences into words, unlike existing tasks (Keating and Cook 2023c, 2023a). This is particularly beneficial here as autistic individuals sometimes have different language and communication profiles to non‐autistic individuals (see Frith and Happé 1994 and Vogindroukas et al. Vogindroukas et al. 2022). Removing the requirement to translate their emotional experiences into words means that our task focuses on participants' ability to differentiate their *own emotional states* (i.e., *their internal emotional reactions*) rather than their ability to produce emotion labels.

The chosen images were known to be effective at selectively evoking anger, happiness, or sadness in large samples of participants (*N* = 124) (Riegel et al. 2016). In this task, we included five images for each emotion (anger, happiness, and sadness) resulting in 15 different images and 105 unique image combinations (and thus 105 trials): 30 within emotion‐category combinations (10 for anger, 10 for happiness, and 10 for sadness) and 75 between emotion‐category combinations (25 angry–sad, 25 angry–happy, 25 happy–sad). To prevent participants from responding too quickly (i.e., without thinking), a reaction time check was incorporated: If participants responded faster than 1000 ms, they were presented with an error message (“Too Fast. Our algorithm has detected that you might need to take longer to think through your answer. You will now incur a 5‐s penalty and then will be asked to do the trial again”), were given a 5‐s penalty, and then the trial re‐started.

To compute the distances between and within emotion clusters, the similarity ratings were transformed into distance scores using multidimensional scaling (with the Scikit‐learn library in Python). This technique allows users to represent objects (here, emotional images) as points in multidimensional space, wherein close similarity between objects corresponds to close distances between the points in the representation (van der Klis and Tellings 2022). To calculate the mean distances within specific emotion clusters, we averaged across the Euclidean distances for the 10 angry–angry, 10 happy–happy, and 10 sad–sad image pairs, respectively. To calculate the mean distance between specific emotion clusters we averaged across the Euclidean distances for the 25 angry–happy, 25 angry–sad, and 25 happy–sad image pairs, respectively. Finally, we computed mean distances within clusters and between clusters by averaging across emotions/emotion pairs. Larger distances between clusters represent a greater ability to differentiate distinct emotional states (e.g., differentiate anger and sadness). Larger distances within clusters represent a greater ability to differentiate similar emotional states (e.g., differentiate irritation from anger).

In the second part of our EmoMap paradigm, on each trial, participants were presented with three images from the Nencki Affective Picture System (Marchewka et al. 2014), and then had to make a decision. This task involved four conditions: one non‐emotional control condition and three emotional experimental conditions assessing the experience of anger, happiness, and sadness respectively. Participants completed the non‐emotional control condition first and then the experimental conditions in a random order. In the control condition, participants had to select which of the three (neutral valence) images they found most colorful using their mouse cursor. On each trial, two of these images were in color and one was in grayscale, thus acting as an attention check. If participants selected the grayscale image, they were presented with the same error message mentioned previously, they incurred a 5‐s penalty, and then had to do the trial again. In the experimental conditions, participants had to select which of the three images made them feel most angry, happy, or sad (e.g., in the angry condition, participants had to decide which of the two images made them most angry) using their mouse cursor. As in the control condition, there was a “trap” image on each trial: Two of the images were strong inducers of the target emotion (e.g., anger), and one was a strong inducer of another emotion (e.g., happiness), thus acting as an attention check. If participants selected the image that strongly induced the non‐target emotion, they were presented with the error message, they incurred a 5‐s penalty, and then had to do the trial again. In each condition, there were 11 target (i.e., non‐trap) images which were presented in all possible unique combinations across 55 trials. The selected images had previously been identified as successful inducers of the target emotion (Riegel et al. 2016).

Consistency scores were calculated for each condition based on the logical consistency of a participant's decisions. To illustrate this, if a participant selects Image A over Image B (A > B) and Image B over Image C (B > C), these decisions are all consistent with one another. However, if the participant then selects Image C over Image A, this would be inconsistent with their previous judgments. Accordingly, consistency requires participants to distinguish between the intensity of emotion evoked by each image across multiple instances, and thus inconsistent decisions are likely to stem from inconsistencies in how individuals experience an emotion.

We followed the procedures outlined previously to calculate emotional consistency (Keating and Cook 2023b; Huggins et al. 2021). We first quantified each participant's image rankings by computing the number of times they chose each image within a condition. If a participant made completely consistent decisions within a condition, rank scores would follow a perfect linear sequence: The image they found most emotionally intense (or colorful) should be chosen in all 10 trials it appeared (score = 10), the second‐highest should be chosen in nine of 10 trials (score = 9), and so on. The image they found least emotionally intense (or colorful) should never be chosen (score = 0). Following this, we examined how image rankings related to the decisions made on each trial. Since images with a higher rank score should elicit a stronger emotional response than those with lower rank scores, we consider an inconsistent decision to be when a lower‐ranking image is chosen over a higher‐ranking image. For each trial, the rank score of the unchosen item was subtracted from the rank score for the chosen item, producing item differences. For consistent decisions, the item difference would be greater than zero; for inconsistent decisions, the item difference would be less than or equal to zero. More severe inconsistencies (e.g., choosing the lowest ranked image over the highest ranked image) result in more negative item differences. Finally, we summed these item differences, per condition, to produce total consistency scores, with greater scores reflecting higher consistency. If a participant made completely consistent decisions within a condition, their score would be 220.

The EmoMap paradigm had strong internal consistency here, with excellent split‐half reliability both for distance scores [distance between cluster: Spearman–Brown coefficient = 0.94; distance within cluster: Spearman–Brown coefficient = 0.90] and emotional consistency scores [Spearman–Brown coefficient = 0.985–0.994 across conditions]. This paradigm also demonstrates very good test–retest reliability, construct validity, and discriminant validity in other work (see Supporting Information C).

#### 3.1.4 PLF Emotion Recognition Task

We assessed participants' emotion recognition performance using the PLF emotion recognition task (Keating, Fraser, et al. 2022; Sowden et al. 2021). In this task, participants viewed dynamic point‐light displays of the face (PLFs), created from videos of four actors saying sentences while posing three target emotions (angry, happy, and sad). These PLFs have been adapted (see Sowden et al. 2021 for further detail) such that they represent three spatial movement levels, ranging from reduced to increased spatial movement (50%, 100%, and 150% spatial movement), and three kinematic levels, ranging from reduced to increased speed (50%, 100%, and 150% original stimulus speed). In this task, each trial began with the presentation of a (silent) PLF video displaying one of the three emotions, at one of the three spatial and three kinematic levels (e.g., Happy at 100% spatial movement and 150% speed). After viewing the PLF stimulus, participants were required to rate how angry, happy, and sad the person was feeling on three visual analogue scales (presented in a random order) ranging from 0, “*not at angry/happy/sad*” to 10, “very angry/happy/sad.” Participants completed three practice trials and then 108 randomly ordered experimental trials (12 per condition) across three blocks. Participants were encouraged to take breaks between blocks.

Emotion recognition accuracy scores were calculated by subtracting the mean of the two incorrect emotion ratings from the correct emotion rating. For instance, for a trial in which a sad PLF was displayed, the mean rating of the two incorrect emotions (angry and happy) was subtracted from the rating for the correct emotion (sad). Mean emotion recognition accuracy was calculated by taking the mean of accuracy scores across all emotions, spatial, and kinematic levels.

The PLF Emotion Recognition task demonstrated excellent reliability, with high internal consistency [Cronbach's *α* = 0.86] and strong split‐half reliability [Spearman–Brown coefficient = 0.92]. This task also demonstrates good test–retest reliability, concurrent validity, construct validity, and discriminant validity in other work (see Supporting Information C).

#### 3.1.5 Emotional Vocabulary Test

We assessed participants' semantic conceptions of 20 different emotions (*affection*, *amusement*, *anger*, *anxiety*, *awe*, *contentment*, *depression*, *desire*, *disgust*, *embarrassment*, *excitement*, *fear*, *guilt*, *happiness*, *interest*, *irritation*, *loneliness*, *peaceful*, *sadness*, *surprise*) using an adapted version of the emotional vocabulary test (from Nook et al. 2017 and Baron‐Cohen et al. 2010). The list of emotions was selected to include (a) the six basic emotions (Ekman and Friesen 1971), (b) emotions that occupy all four quadrants of the circumplex dimensions of arousal and valence (Russell 1980), and (c) emotions that are most frequently evoked by standardized databases of images (e.g., the Nenki Affective Picture System, the International Affective Picture System) (Mikels et al. 2005). In this task, on each trial, participants were required to type a definition of an emotion word that was presented on screen. To ensure data validity, we (i) explicitly instructed participants to come up with definitions themselves (rather than searching for them online), (ii) forced the task into full‐screen so that we could tell if participants minimized the page to look‐up definitions, and (iii) excluded any definitions that matched those provided by the Oxford, Cambridge, and Meriam Webster dictionaries.

In the current study, we consider how well participants understand the meaning (i.e., the semantic content) of emotion concepts, and how these meanings overlap between emotions. To this end, we calculated two types of scores using the definitions provided by participants—emotional vocabulary test scores, which pertain to the accuracy of participants' definitions, and conceptual distance scores, which reflect the conceptual overlap in participants' own definitions. To calculate emotional vocabulary scores, first, a trained experimenter assigned each definition a score of zero, one, or two (as in a WASI vocabulary test and in Nook et al. 2017). A score of two was awarded if the participants provided (i) a plausible and specific definition of the emotion, (ii) a direct synonym of the emotion, or (iii) a scenario that would conceivably evoke the given emotion and no other emotions. We assembled a list of definitions and synonyms (taken from the Oxford and Cambridge Dictionaries and from Nook et al. 2017) which the experimenter referred to when scoring the responses. A score of one was awarded if the participant provided a definition that was of the correct valence or situation, but too vague to meet criteria for a two‐point response. For example, if a participant defined loneliness as “the feeling of being alone” or “a sad feeling”, they would score one point for this definition. To score two points, participants would need to include both parts of this definition: for example, “the sad feeling you get when you are alone”. A score of 0 was awarded if the participants gave definitions, synonyms, or situations relevant to a different emotion. We calculated total emotional vocabulary scores by summing the scores for each item. As such, emotional vocabulary test scores ranged from 0 to 40, with higher scores representing more accurate understanding of emotion terms.

To calculate conceptual distance scores, we employed Natural Language Processing—a machine learning technique facilitating the analysis and synthesis of large quantities of language data (Khurana et al. 2023). Specifically, we used a pre‐existing model (*sentence‐transformers/all‐mpnet‐base‐v2*) designed to analyze the meaning of sentences, and then compute the conceptual similarity of sentence pairs (i.e., the similarity in meaning of sentence pairs). During its development, this model was trained on one billion sentence pairs, derived from numerous online sources, thus enhancing the reliability of the conceptual similarity estimates. In the current study, we used this model to compute conceptual similarity scores for each pair of definitions (e.g., affection–amusement, affection–anger, affection–anxiety… sadness–surprise), which we then inverted (by multiplying by −1) to get conceptual distance scores. These conceptual distance scores range from 0 to −1 (to 15 decimal places), with higher scores representing greater differentiation of semantic emotion concepts. To assess the differentiation of participants' conceptions of same‐valence emotions (within valence conceptual distance), we took a mean of the conceptual distance scores for the 45 positive–positive definition pairs (e.g., affection–amusement, affection–happiness, etc.), and 45 negative–negative definition pairs (e.g., anger–anxiety, anger–sadness, etc.), and then averaged across these values. To assess the differentiation of participants' conceptions of opposite‐valence emotions (between valence conceptual distance), we took a mean of the conceptual distance scores for the 100 positive–negative definition pairs.

In the current study, the Emotional Vocabulary Test had strong internal consistency and split‐half reliability for total test scores [Cronbach's *α* = 0.858; Spearman–Brown coefficient = 0.878] and conceptual distance scores [Cronbach's *α* = 0.986; Spearman–Brown coefficient = 0.943]. This task also demonstrates good construct validity and discriminant validity in other work (see Supporting Information C).

Logically, if our task and analysis pipeline are operating as intended, between valence conceptual distance scores should be higher (i.e., more positive) than within valence conceptual distance scores. In order to verify this, we conducted a paired samples t‐test on these data, identifying *extreme* evidence [BF_10_ > 100] that between valence conceptual distance scores [mean (SEM) = −0.28 (0.006)] were higher than within valence conceptual distance scores [mean (SEM) = −0.40 (0.008); *t*(99) = 32.55, *p* < 0.0001, BF_10_ = 1.34e^51^]. Encouragingly, we also identified that the five lowest mean conceptual distance scores were for the anxiety and fear [mean (SEM) = −0.59 (0.015)], depression and sadness [mean (SEM) = −0.58 (0.017)], Contentment and Peaceful [mean (SEM) = −0.56 (0.020)], contentment and happiness [mean (SEM) = −0.55 (0.019)], and Anger and Irritation [mean (SEM) = −0.54 (0.018)] definition pairs, as one would expect (as these concepts are close to one another in meaning).

In addition, we calculated the mean number of words included across all definitions for each participant. This variable was included as a covariate to control for overall definition length, ensuring that emotional vocabulary score, between‐valence conceptual distance, and within valence conceptual distance related to differences in definition content rather than differences in how much participants wrote. That is, including mean definition word count as a covariate allowed us to test whether these conceptual definition measures predicted outcomes above and beyond the amount of text participants provided.

#### 3.1.6 The Matrix Reasoning Item Bank

Participants' non‐verbal reasoning ability was assessed via the Matrix Reasoning Item bank (see MaRs‐IB) (Chierchia et al. 2019). Each item in the MaRs‐IB consists of a three‐by‐three matrix; eight of the nine available cells are filled with abstract shapes, and one cell is left empty. Participants are required to complete the matrix by selecting the missing shape from four options. To provide the correct answer, participants must deduce relationships between the shapes in the matrix (which vary in shape, color, size and position). After selecting an answer, the participants proceed to the next trial. If they do not provide a response within 30 s, they proceed to the next trial without a response. This assessment lasts eight min regardless of how many trials are completed. The MaRs‐IB has acceptable internal consistency (Kuder–Richardson 20 ≥ 0.7) and test–retest reliability (*r* ≥ 0.7) (Chierchia et al. 2019). Non‐verbal reasoning scores are calculated as the percentage of correct answers across all trials.

### 3.2 Statistical Analyses

All frequentist analyses were conducted using *R Studio* (version 2021.09.2) and all Bayesian analyses were conducted using *JASP* (version 0.16). For all frequentist analyses, we used a significance threshold of *p* = 0.05 (two‐sided) to determine whether to accept or reject the null hypothesis. Parametric assumptions were met for all analyses employing simple linear models and linear mixed effects models. Non‐parametric linear regressions were conducted when assumptions were violated. We conducted all linear mixed effects models in R Studio using the lmer function (from the *lme4* package). In addition, we employed the Anova function (from the *car* package) to conduct a Type III ANOVA on the results of our linear mixed model with a Kenward and Roger (1997) approximation for degrees of freedom, as supported by Luke (2017). In R Studio, we also conducted (i) a random forest analysis (Breiman 2001) employing the Boruta wrapper algorithm (Boruta function from *Boruta* package (Kursa and Rudnicki 2010)), and (ii) mediation analyses using the sem() function (from the *lavaan* package). We conducted Bayesian analyses in JASP in order to determine the relative strength of evidence for the experimental versus null hypotheses. For all Bayesian analyses, we followed the classification scheme proposed by Lee and Wagenmakers (2014), in which BF_10_ and BF_01_ values between one and three reflect weak evidence, between 3 and 10 reflect moderate evidence, greater than 10 reflect strong evidence, and greater than 100 reflect extreme evidence for the *experimental* (BF_10_) and *null* (BF_01_) hypotheses, respectively.

## 4 Results

In the following section, we (1) compare autistic and non‐autistic participants on the consistency and differentiation of emotional experiences, understanding of emotion concepts, and differentiation of emotion concepts, and (2) determine whether the same processes are implicated in autistic and non‐autistic emotion recognition.

### 4.1 Analyses Comparing Autistic and Non‐Autistic Participants

#### 4.1.1 No Differences Between Groups in Emotional Consistency

First, to compare the consistency of emotional experiences across participant groups, we conducted a linear mixed effects model with emotional consistency as the dependent variable, emotion (angry, happy, sad), group (autistic, non‐autistic), the interaction between emotion and group [independent variables], age, sex, non‐verbal reasoning ability, years of education, alexithymia, emotional vocabulary score, between valence conceptual distance, within valence conceptual distance, and mean definition word count [control variables] as predictors, and subject number as a random intercept. This revealed that within valence conceptual distance [*F*(1, 105) = 5.57, *p* = 0.020] and years of education [*F*(1, 105) = 4.19, *p* = 0.043] were positive predictors of emotional consistency: those with more differentiated conceptions of same‐valence emotions, and those with more years of education, typically had greater emotional consistency. There were no other significant predictors [all *p* > 0.05]. Most notably, there was no main effect of group [*F*(1, 284.67) = 0.39, *p* = 0.533], and no emotion × group interaction [*F*(2, 230) = 0.43, *p* = 0.649]. To assess the strength of evidence for these null effects, we conducted a post hoc Bayesian ANOVA. This yielded moderate evidence against a main effect of group (BF_01_ = 5.37) and strong evidence against an emotion × group interaction (BF_01_ = 11.65), suggesting no differences in emotional consistency between autistic and non‐autistic participants, across all three emotions.

#### 4.1.2 No Differences Between Groups in Emotion Differentiation for Distinct Emotional States

To test whether autistic adults have less differentiated experiences of distinct emotions than non‐autistic adults, we constructed a linear mixed effects model with distance between clusters as the dependent variable, emotion pair (angry–happy, angry–sad, happy–sad), group (autistic, non‐autistic), the interaction between emotion pair and group [independent variables], age, sex, non‐verbal reasoning, years of education, alexithymia, emotional vocabulary score, within valence conceptual distance, between valence conceptual distance, and mean definition word count [control variables] as predictors, and subject number as a random intercept. In line with the results from our previous study (Keating and Cook 2023b), there was a significant main effect of emotion pair [*F*(2, 230) = 82.81, *p* < 0.0001]: The distance between angry and sad clusters was smallest [mean (SEM) = 13.78 (0.28)], followed by happy and sad [mean (SEM) = 17.64 (0.45)], followed by angry and happy [mean (SEM) = 18.46 (0.45)]. In addition, between valence conceptual distance [*F*(1, 105) = 5.13, *p* = 0.026] was a significant positive predictor of distance between clusters: Those with less differentiated conceptions of emotions (of opposite valence) typically had less differentiated *experiences* of distinct emotions. Finally, our analysis also revealed that non‐verbal reasoning ability was a significant negative predictor of distance between clusters [*F*(1, 105) = −6.83, *p* = 0.010]: those with higher non‐verbal reasoning ability typically had smaller distances between clusters. Once again there was no main effect of group [*F*(1, 143.04) = 2.50, *p* = 0.116], nor an interaction between emotion pair and group [*F*(2, 230) = 1.68, *p* = 0.189], and no other significant predictors of distance between clusters [all *p* > 0.05]. To evaluate the strength of evidence for these null effects, we conducted a follow‐up Bayesian ANOVA. There was anecdotal evidence against a main effect of group (BF_01_ = 1.24) and moderate evidence against an emotion × group interaction (BF_01_ = 3.98), suggesting there are no differences between the autistic and non‐autistic participants in the differentiation of distinct emotional states.

#### 4.1.3 No Differences Between Groups in Emotion Differentiation for Similar Emotional States

Next, to test whether autistic adults have less differentiated experiences of similar emotions (i.e., less granular emotional experiences), we constructed a linear mixed effects model with distance within clusters as the dependent variable, emotion (distance within angry, happy, and sad clusters, respectively), group (autistic, non‐autistic), the interaction between emotion and group [independent variables], age, sex, non‐verbal reasoning, years of education, alexithymia, emotional vocabulary score, within valence conceptual distance, between valence conceptual distance, and mean definition word count [control variables] as predictors, and subject number as a random intercept. This revealed a main effect of emotion [*F*(2, 230) = 15.52, *p* < 0.001]: Distance within happy clusters was lowest [mean (SEM) = 12.30 (0.31)], followed by distance within angry clusters [mean (SEM) = 13.57 (0.26)], and distance within sad clusters [mean (SEM) = 13.65 (0.30)]. In addition, our analysis identified that between valence conceptual distance [*F*(1, 105) = 5.40, *p* = 0.022] was a significant positive predictor of distance within clusters: those with less differentiated conceptions of emotions (of opposite valence) typically had less differentiated experiences of similar emotions. We also identified that non‐verbal reasoning was a significant negative predictor of distance within clusters [*F*(1, 105) = 15.32, *p* < 0.001]. There was no main effect of group [*F*(1, 144.57) = 0.36, *p* = 0.549], nor an emotion × group interaction [*F*(2, 230) = 1.12, *p* = 0.327], and there were no other significant predictors of distance within clusters [all *p* > 0.05]. To probe the strength of evidence for these null effects, we conducted a follow‐up Bayesian ANOVA. There was anecdotal evidence against a main effect of group (BF_01_ = 2.60) and moderate evidence against an emotion × group interaction (BF_01_ = 6.67), suggesting there are no differences between the autistic and non‐autistic participants in the differentiation of similar emotional states.

#### 4.1.4 No Differences Between Groups in Levels of Understanding of Emotion Concepts

To assess the understanding of emotion concepts, we compared the emotional vocabulary test scores of the autistic and non‐autistic participants. To do so, we ran a non‐parametric multiple regression of emotional vocabulary as a function of group (autistic, non‐autistic), age, sex, non‐verbal reasoning, years of education, alexithymia, and mean definition word count [control variables]. This analysis revealed that mean definition word count [*t*(108) = 3.63, *p* < 0.001] was a positive predictor, and age [*t*(108) = −2.90, *p* = 0.005] was a negative predictor of emotional vocabulary score: those who provided longer definitions, and those younger in age, typically had higher emotional vocabulary scores. There were no significant differences between the autistic participants and non‐autistic participants in emotional vocabulary score [*t*(108) = −1.64, *p* = 0.104]. A follow‐up Bayesian independent sample t‐test revealed anecdotal evidence for this null effect [BF_01_ = 2.49]. Together, our results suggest that there are no differences between groups in the understanding of emotion concepts.

#### 4.1.5 No Differences Between Groups in the Differentiation of Semantic Emotion Concepts

Following this, to determine whether autistic or non‐autistic people have more differentiated conceptions of emotions with the same and opposite valences, we constructed two simple linear models as a function of group (autistic, non‐autistic), age, sex, non‐verbal reasoning, years of education, alexithymia, and mean definition word count [control variables]. Across both models, mean definition word count was a negative predictor [between valence: *F*(1, 108) = −10.30, *p* = 0.002; within valence: *F*(1, 108) = −11.66, *p* < 0.001] and age [between valence: *F*(1, 108) = 5.18, *p* = 0.024; within valence: *F*(1, 108) = 8.33, *p* = 0.005] was a positive predictor: those who provided longer definitions, and those younger in age, tended to have lower conceptual distance scores, both for same‐valence and opposite‐valence emotions. There was no effect of group [between valence conceptual distance: *F*(1, 108) = 1.00, *p* = 0.320; within valence conceptual distance: *F*(1, 108) = 3.13, *p* = 0.080], nor any other significant predictors in both models [all *p* > 0.05]. In our follow‐up Bayesian independent samples t‐tests, there was moderate evidence for a null effect of group for both between valence conceptual distance [BF_01_ = 5.05] and within valence conceptual distance [BF_01_ = 3.66]. Together, the evidence suggests that there were no differences in the differentiation of semantic emotion concepts between autistic and non‐autistic participants.

In sum, we found no credible evidence for differences between autistic and non‐autistic individuals in emotional consistency, the differentiation of emotional experiences, or the understanding and differentiation of semantic emotion concepts, after controlling for alexithymia. Notably, in our sample, 15 of the non‐autistic participants scored above cut‐off for potential autism on the AQ (≥ 26) while six autistic participants scored below it (< 26). To ensure that the absence of significant group differences was not due to high autistic traits in the non‐autistic group and low traits in the autistic group—which could reduce between‐group differences—we excluded these participants and repeated our analyses. The pattern of results was unaffected by these exclusions; there were still no differences between the autistic and non‐autistic participants on our variables of interest (see Supporting Information D).

### 4.2 Different Combinations of Variables Are Important for Autistic and Non‐Autistic Emotion Recognition

Next, we aimed to determine the factors contributing to autistic and non‐autistic emotion recognition via random forests analyses (Breiman 2001) with the Boruta wrapper algorithm (Lee and Wagenmakers 2014) (version 7.7.0; as in Keating and Cook 2023b and Keating et al. 2023). Across numerous iterations (here, 3000), this algorithm trains a random forest regression model on all predictor variables, as well as their permuted copies (known as “shadow features”), and classifies a variable as important (i.e., useful for predicting a target variable) when its importance score is higher than the maximum score amongst all shadow features (termed “shadowMax” in the analysis; see Mazzanti 2020 for an accessible summary of the Boruta wrapper algorithm). In this analysis, our outcome variable was mean emotion recognition accuracy. Predictors included the emotion‐related variables examined in this study: emotional consistency, distance between clusters, distance within clusters, emotional vocabulary score, within‐valence conceptual distance, and between‐valence conceptual distance. For exploratory purposes, we also included total AQ score, total TAS score, the AQ and TAS subscales (i.e., AQ social skills, AQ attention switching, AQ attention to detail, AQ communication, AQ imagination, TAS difficulties describing feelings, TAS difficulties identifying feelings, and TAS externally oriented thinking), non‐verbal reasoning ability, years of education, and age as predictors (thus following similar procedures to Keating and Cook 2023b and Keating et al. 2023), since these variables are also thought to be involved in emotion‐processing (Keating and Cook 2020, 2023b; Keating, Fraser, et al. 2022; Rump et al. 2009).

**FIGURE 1:**
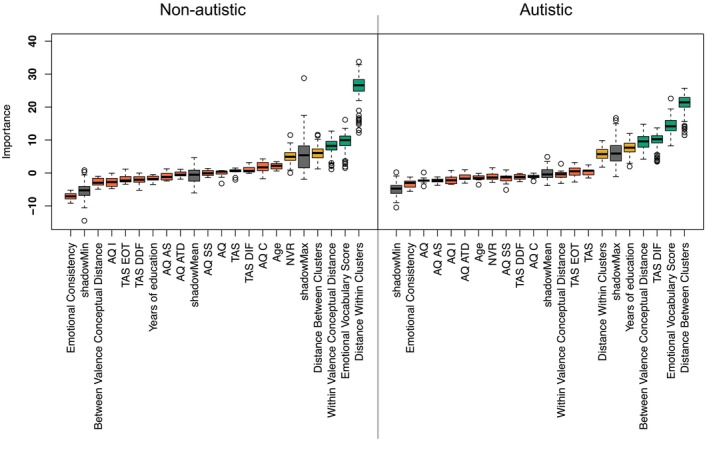
Random forest variable importances for non‐autistic (left) and autistic (right) participants. Variable importance of all 19 features entered into the Boruta random forest, displayed as boxplots. Box edges denote the interquartile range (IQR) between the first and third quartile; whiskers denote 1.5*IQR distance from box edges; circles represent outliers outside of 1.5*IQR above and below box edges. Box color denotes decision: Green—confirmed, yellow = tentative, red = rejected; gray = meta‐attributes shadowMin, shadowMax and shadowMean (minimum, maximum and mean variable importance attained by shadow features).

For the non‐autistic participants, of the 19 variables tested, three were classified as important, two were classified as tentatively important, and 14 were deemed unimportant. Figure 1 (left) illustrates that the distance within clusters [mean importance score (MIS) = 25.70], emotional vocabulary score [MIS = 9.43], and within valence conceptual distance [MIS = 7.89] were important for emotion recognition; distance between clusters [MIS = 6.06] and non‐verbal reasoning [MIS = 5.09] were tentatively important for emotion recognition. All other variables were deemed unimportant.

In comparison, for the autistic participants, four of the variables were classified as important, two tentatively important, and the remainder unimportant for emotion recognition. Figure 1 (right) shows that the distance between emotion clusters [MIS = 20.80], emotional vocabulary score [MIS = 14.31], the TAS difficulty identifying feelings subscale [MIS = 9.86], and between valence conceptual distance [MIS = 9.26] were deemed important; years of education [MIS = 7.56] and distance within clusters [MIS = 5.71] were tentatively important for emotion recognition. All other variables were classified as unimportant.

**FIGURE 2:**
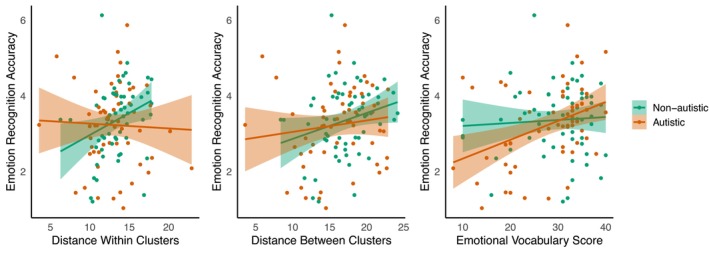
The relationships between mean emotion recognition accuracy and distance between clusters, distance within clusters, and emotional vocabulary score, respectively, for the autistic (orange) and non‐autistic (green) participants.

Next, to verify the results from our random forests regression model, we constructed linear mixed effects models predicting mean emotion recognition accuracy with the important and tentatively important variables in the autistic and non‐autistic groups respectively. Since we identified a strong correlation between two variables of interest—distance between emotion clusters and distance within clusters [*R* = 0.851, *p* < 0.001]—we constructed two linear mixed effects models with near identical predictors but where one model included distance *between* emotion clusters and the other included distance *within* clusters. Thus, ensuring that parameter estimates were not compromised by collinearity issues (Johnston et al. 2018). In the model that excluded distance *between* clusters, distance within clusters [*F*(1, 54) = 8.55, *p* = 0.005] and non‐verbal reasoning [*F*(1, 54) = 4.32, *p* = 0.042] were significant positive predictors of non‐autistic emotion recognition. In the model that excluded distance *within* clusters, distance between clusters was a significant positive predictor of emotion recognition [*F*(1, 54) = 4.84, *p* = 0.032]. In sum, non‐autistic individuals who can more accurately distinguish between similar and distinct emotional states (see Figure 2) tend to excel in emotion recognition, aligning with previous findings (Keating and Cook 2023b).

Following this, we constructed the relevant linear mixed effects models in the autistic group. In the model that excluded distance *between* clusters, emotional vocabulary score was a significant positive predictor of autistic emotion recognition [*F*(1, 52) = 5.31, *p* = 0.025]. There were no other significant predictors [all *p* > 0.05]. In the model that excluded distance *within* clusters, again emotional vocabulary score was the only significant predictor of emotion recognition for autistic people [*F*(1, 52) = 5.36, *p* = 0.025]. Thus, for autistic people, having a clear understanding of emotion concepts is linked to more accurate emotion recognition.

In sum, while being able to differentiate similar and distinct emotional states was linked to enhanced emotion recognition for non‐autistic individuals, having a greater understanding of emotion concepts predicted elevated emotion recognition for autistic people.

### 4.3 Exploratory Analyses: Emotion Differentiation Mediates the Relationship Between the Differentiation of Semantic Emotion Concepts and Emotion Recognition for Non‐Autistic People

Since we had identified that between valence conceptual distance predicted distance between and within clusters, which both predicted emotion recognition performance for non‐autistic people, we conducted post hoc mediation analyses to explore whether between valence conceptual distance exerted an indirect effect on emotion recognition by influencing the distances between and within clusters. Here, we used structural equation modeling (SEM) for our mediation analyses, rather than standard regression models (Baron and Kenny 1986), as SEM is regarded “a more appropriate inference framework for mediation analyses” (Gunzler et al. 2013). We employed bias‐corrected bootstrapping (with 2000 replications) to create 95% confidence intervals (95% CI) as there is a consensus that this is the most powerful method for testing mediated effects (Cheung 2007; Fritz and MacKinnon 2007; MacKinnon et al. 2004; Valente et al. 2016). If these confidence intervals do not cross zero, there is evidence for the experimental hypothesis; if these confidence intervals cross zero, there is evidence for the null hypothesis (Foster et al. 2018).

In the first model, the predictor was between valence conceptual distance, the mediator was distance *between* clusters, and the outcome variable was emotion recognition accuracy. In the second model, the predictor was between valence conceptual distance, the mediator was distance *within* clusters, and the outcome variable was emotion recognition. Across all mediation models we controlled for relevant confounding variables (e.g., non‐verbal reasoning, AQ, TAS, years of education, emotional vocabulary score, mean definition word count) to enhance the internal validity of our findings.

**FIGURE 3:**
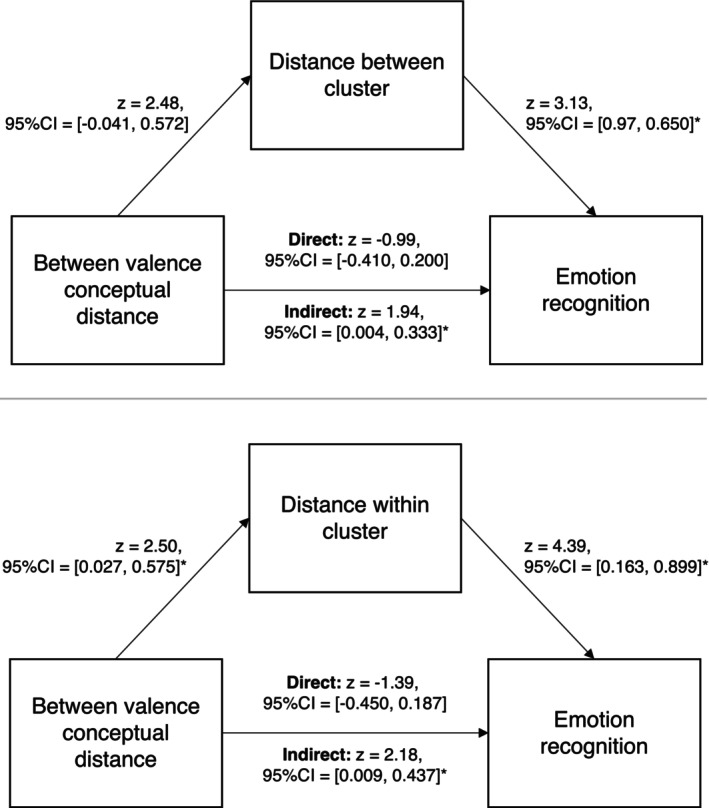
Mediation models showing the contribution of between valence conceptual distance to *non‐autistic* emotion recognition via distance between clusters (top) and distance within clusters (bottom). The asterisks (*) denote statistical significance according to confidence intervals.

In the first model, although there was no direct effect [*z* = −0.99, 95% CI = (−0.410, 0.200)] of between valence conceptual distance on emotion recognition, there was an indirect effect via distance between clusters [*z* = 1.94, 95% CI = (0.004, 0.333); see Figure 3, top]. This suggests a potential causal direction (though future studies are necessary to confirm this chain of causality); for non‐autistic people, having well‐differentiated conceptions of emotion may lead to individuals having well‐differentiated experiences of distinct emotions, and then in turn greater emotion recognition accuracy. Similarly, in the second model, there was no direct effect of between valence conceptual distance on emotion recognition [*z* = −1.39, 95% CI = (−0.450, 0.187)], but there was an indirect effect via distance within clusters [*z* = 2.18, 95% CI = (0.009, 0.437); see Figure 3, bottom.]. As such, for non‐autistic participants, having well‐differentiated conceptions of emotion may also lead to them having well‐differentiated experiences of similar emotions, and then in turn greater emotion recognition accuracy. Future studies employing causal manipulation are needed to confirm these chains of causality.

To verify that these pathways were most plausible, we then swapped the position of distance between clusters and between valence conceptual distance, such that distance between clusters was the predictor and between‐valence conceptual distance was the mediator. Our analysis revealed that the indirect effect was not significant [*z* = −0.92, 95% CI = (−0.232, 0.034)]. Following this, we conducted the same analysis with distance within clusters, identifying once again that the indirect effect was not significant [*z* = −1.22, 95% CI = (−0.273, 0.031)]. Therefore, our results suggest that the most mathematically plausible pathway is as follows: having well differentiated conceptions of emotion may lead to more differentiated experiences of emotion, and then in turn better emotion recognition.

## 5 Discussion

The current study compared autistic and non‐autistic adults on emotional abilities thought to be involved in emotion recognition (e.g., emotional consistency, differentiation of experiences and concepts of emotion, and understanding of emotion concepts), and investigated the contribution of these factors to emotion recognition in both groups. Our results provide no credible evidence for differences between autistic and non‐autistic adults with respect to the consistency and differentiation of emotional experiences, nor the understanding or differentiation of semantic concepts of emotion. This held true across both complex statistical models (see Section 4) and simpler models with fewer predictors (see Supporting Information E), and in both the full sample and a more conservative subsample (excluding non‐autistic participants who scored above the cutoff on the AQ and autistic participants who scored below it; see Supporting Information D). This convergence across models and samples strengthens our confidence in the finding that autistic and non‐autistic individuals do not differ on these emotion‐related factors. However, notably, we identified differences in the traits, processes, and abilities involved in autistic and non‐autistic emotion recognition. Although for non‐autistic individuals, having more differentiated conceptualizations and experiences—for distinct (e.g., angry–happy, angry–sad, happy–sad) and similar (e.g., anger, irritation, frustration) emotions—was linked to enhanced emotion recognition, having a more precise understanding of emotion concepts (as indexed by more precise definitions of emotion terms) predicted emotion recognition for autistic people.

These findings significantly deepen our understanding of the processes and abilities involved in both autistic and non‐autistic emotion recognition. To the best of our knowledge, no studies to date have empirically tested the mechanistic pathway by which emotion concepts influence emotion recognition. As discussed in the Introduction, one possibility is that emotion concepts impact upon emotional experiences and emotion perceptions directly and independently; another possibility is that there are indirect effects among these variables. The current study suggests that the latter is more mathematically plausible; having well‐differentiated *concepts* of emotion *may* lead to (non‐autistic) individuals having well‐differentiated *experiences* of emotion, and then in turn greater emotion recognition accuracy. This chain of causality raises a hypothetical pathway by which these abilities develop from infancy to adulthood (i.e., emotion concepts become increasingly differentiated, leading to increasingly differentiated emotional experiences, and then in turn emotion perceptions). Nevertheless, further research employing causal manipulation and/or longitudinal methods is necessary to verify this chain of causality, and to test how and when these links arise developmentally. For example, future studies could assess our emotion‐related variables at baseline and then again following one of two interventions: (1) targeted training designed to improve participants' ability to differentiate the meanings of various emotions, and (2) an active control intervention focused on improving the ability to differentiate various colors. If those in the emotion‐focused intervention show greater improvements than those in the control condition—not only in differentiating semantic emotion concepts, but also in distinguishing their own or others' emotions—this would provide evidence for a causal link between these abilities. To further explore causality, researchers could examine whether the extent of improvement in differentiating semantic emotion concepts is associated with improvements in emotion differentiation or emotion recognition. If these associations are found, this would provide further evidence that enhancing the differentiation of semantic emotion concepts leads to downstream gains in emotion differentiation and recognition.

Similarly, these findings also significantly advance our understanding of the abilities and processes involved in autistic emotion recognition. Until now, the factors involved in autistic emotion recognition have remained elusive, with several studies finding that certain demographic factors, abilities, or processes important for non‐autistic emotion recognition are not important for autistic emotion recognition (Brewer et al. 2016; Rump et al. 2009; Keating et al. 2023; Keating, Sowden, et al. 2026). This has led to arguments that autistic people may adopt alternative, cognitively mediated strategies to recognize the emotions of other people (Rutherford and McIntosh 2007; Walsh et al. 2014). If this were true, one might expect a stronger link between emotion recognition performance and cognitive ability in autistic individuals compared to non‐autistic individuals. Supporting this, previous work has found that IQ (Keating, Sowden, et al. 2026) and mental age (Hobson 1986) are linked to enhanced emotion recognition performance for autistic but not non‐autistic people. Building on these findings, our study found that a stronger *understanding* of emotion terms was linked to enhanced emotion recognition for autistic people only. This lends further support to the idea that autistic people may follow alternative, cognitively mediated strategies to recognize the emotions of other people.

The results of the current study contradict previous findings suggesting that autistic individuals have less differentiated experiences and concepts of emotions (Erbas et al. 2013). There are numerous potential explanations for this discrepancy. First, in the analyses conducted here, we have controlled for alexithymia—an important confounding variable that was not controlled for in previous studies. Hence, it is possible that the autistic participants tested in previous studies had less differentiated experiences and concepts of emotion due to co‐occurring alexithymia, rather than due to autism itself, in line with the alexithymia hypothesis (Bird and Cook 2013). Second, it could be the case that autistic individuals have particular difficulties on emotion differentiation tasks that require them to translate their emotional experiences into words (e.g., the photo emotion differentiation task in Erbas et al. 2013), but do not exhibit differences with respect to tasks that purely focus on differentiating internal emotional states (such as our EmoMap task). Although this thesis is a possibility, it is not probable since, if this were the case, we would have expected our autistic participants to perform more poorly than their non‐autistic counterparts on the emotional vocabulary test. Third, this discrepancy in findings could arise due to differences in demographics. Although the sample in the current study comprised 58 autistic and 59 non‐autistic *adults* (with a mean age in each group of 33.26 and 32.27 years respectively), previous studies have tested younger samples (e.g., Erbas et al. (2013) tested 18 autistic and 26 non‐autistic *adolescents* with a mean age of 16.71 and 16.56 years, respectively). As such, it is possible that autistic individuals have particular difficulties differentiating experiences and concepts of emotions relative to their non‐autistic peers during adolescence, which disappear as they transition into adulthood. Further research is necessary to (a) replicate the results of the current study, and (b) formally test under what conditions and tasks (e.g., age, language‐based tasks) autistic people exhibit difficulties with emotion differentiation.

### 5.1 Implications

The results of the current study pave the way for future support systems to help *both* autistic and non‐autistic people to accurately recognize emotional facial expressions. Specifically, such systems could support non‐autistic individuals in *distinguishing* emotional experiences and concepts while helping autistic individuals deepen their *understanding* of emotion concepts—which could enhance emotion recognition in both groups. Indeed, recent work demonstrates the promise of this approach. This study used a five‐day intervention to deepen participants' understanding of emotion concepts and improve their ability to differentiate them by providing detailed information and encouraging comparisons between concepts (Vedernikova et al. 2021). This intervention had promising effects, successfully improving emotion concept knowledge and downstream emotion differentiation performance, relative to an active control group, immediately after training and at follow‐up a month later (Vedernikova et al. 2021). Such interventions have the potential for widespread benefits: Improved emotion differentiation is linked not only to accurate emotion recognition but also to adaptive emotion regulation, better psychosocial functioning, and reduced mental health difficulties (Smidt and Suvak 2015; Kashdan et al. 2015; Trull et al. 2015; Hoemann et al. 2021; Thompson et al. 2021; O'Toole et al. 2020; Seah and Coifman 2022). Further research is necessary to assess whether such interventions have longer term benefits for conceptual emotion knowledge and emotion differentiation, and to determine whether these interventions could have downstream benefits for autistic and non‐autistic emotion recognition.

### 5.2 Limitations and Future Directions

It is important to address the limitations of our study with respect to sample generalizability. The participants in the current study were predominantly White (78.6%; see Supporting Information A), highly educated (47% with an undergraduate bachelor's degree or higher; see Supporting Information B), adults from the United Kingdom. As such, our sample may not be representative of those with lower levels of education or intellectual disabilities, or those from different racial, ethnic, or cultural backgrounds. While the inclusion of adults is a significant strength of this study, as this group is typically greatly underrepresented in autism research (just 21% of studies involve this group (Kirby and McDonald 2021)), it is important to note that our findings may not generalize to children and adolescents. As discussed, there may be differences between autistic and non‐autistic individuals in emotion‐processing during childhood and adolescence that disappear as they transition into adulthood. Finally, in the current study, our sample comprised a slightly higher number of females than males in both the autistic (53% female, 41% male) and non‐autistic (56% female, 44% male) groups. This is also a strength of the study, as women are often underrepresented in autism research (Watkins et al. 2014; Mo et al. 2021). However, since modern estimates indicate that there are two males to every one autistic female (D'Mello et al. 2022), our sample may not be representative of the autistic population more generally. Nevertheless, our post hoc tests demonstrate that there were no interactions between sex and group in our analyses comparing the autistic and non‐autistic individuals on emotion‐processing (see Supporting Information F). These results suggest that there are no differences between autistic and non‐autistic adults in emotional consistency, emotion differentiation, and the understanding or differentiation of emotion concepts, irrespective of sex. As such, our findings are likely to be representative of both males and females.

Finally, although here we found no significant differences between autistic and non‐autistic adults on our emotion‐related variables, our Bayesian analyses only found anecdotal‐moderate evidence for these null effects. Specifically, we found anecdotal evidence that there are no differences between groups with respect to emotion differentiation [distance between clusters: BF_01_ = 1.24; distance within clusters: BF_01_ = 2.60] and the understanding of emotion terms [BF_01_ = 2.49], and moderate evidence with respect to emotional consistency [BF_01_ = 5.37] and the differentiation of semantic emotion concepts [between valence conceptual distance: BF_01_ = 5.05; within valence conceptual distance: BF_01_ = 3.66]. While the moderate evidence for these latter effects affords us confidence in our findings, future investigations should aim to replicate these results, and particularly those regarding emotion differentiation and the understanding of emotion terms. As this study was not pre‐registered, incorporating pre‐registered protocols in future work would further strengthen external confidence in our results. Consistent with our approach, such future investigations should control for alexithymia to ensure that any differences between groups truly arise due to autism, rather than due to underlying alexithymia.

### 5.3 Conclusions

This study found no differences between autistic and non‐autistic adults in the consistency or differentiation of their emotional experiences, nor their understanding or differentiation of semantic emotion concepts, after accounting for alexithymia. However, there were differences in the psychological mechanisms involved in autistic and non‐autistic emotion recognition. For non‐autistic people, the ability to differentiate one's own emotions contributed to enhanced emotion recognition. Although having more differentiated emotion concepts (indirectly) contributed to elevated emotion recognition for non‐autistic people, having a more precise understanding of emotion concepts contributed for autistic people. The results of the current study pave the way for future systems to help both autistic and non‐autistic people to more accurately recognize emotional facial expressions.

## Author Contributions

C.T.K. and J.L.C. conceptualized and designed the study. C.T.K. and C.K. collected the data. C.T.K. and C.K. analyzed the data. C.T.K. wrote an initial draft. Supervision was conducted by J.L.C. All authors reviewed and provided feedback on the draft and approved the submitted manuscript.

## Funding

This project was supported by the Medical Research Council (MRC, United Kingdom) (MR/R015813/1) and the European Union's Horizon 2020 Research and Innovation Programme under ERC‐2017‐STG (grant agreement no. 757583).

## Ethics Statement

This study was approved by the Science, Technology, Engineering and Mathematics (STEM) ethics committee at the University of Birmingham (ERN_16‐0281AP9D) and conducted in line with the principles of the revised Helsinki Declaration.

## Consent

All participants provided informed consent.

## Conflicts of Interest

The authors declare no conflicts of interest.

## Supporting information


**Data S1:** aur70162‐sup‐0001‐supinfo.docx.

## Data Availability

The data and analysis script corresponding to this study are available online at https://osf.io/4xfet/. Many of the tasks used in the current study are also openly available online at https://app.gorilla.sc/openmaterials/447800.
