# Supplementary material for: The Conceptualization, Experience, and Recognition of Emotion in Autism: Differences in the Psychological Mechanisms Involved in Autistic and Non‐Autistic Emotion Recognition
Source: Autism Res. 2026 Jan 25;19(2):e70162. doi: 10.1002/aur.70162 (PMC12948658; doi:10.1002/aur.70162)
Supplement: Supplementary file 1 — Data S1: aur70162‐sup‐0001‐supinfo.docx. [file AUR-19-0-s001.docx]

**Supporting Information**

**The conceptualisation, experience, and recognition of emotion in autism: Differences in the psychological mechanisms involved in autistic and non-autistic emotion recognition**

Connor T. Keating, Carmen Kraaijkamp, Jennifer L. Cook

**Supporting Information A**

***Table S1.*** Participants’ self-reported ethnicities.

| **Ethnicity** | **N** |
| --- | --- |
| Asian Bangladeshi | 2 |
| Asian British | 4 |
| Asian Indian | 3 |
| Asian Iranian | 1 |
| Asian Sri Lankan | 1 |
| Black African | 3 |
| Black and Indian African | 1 |
| Black British | 2 |
| Chinese | 1 |
| Southeast Asian | 1 |
| Turkish | 1 |
| White American | 1 |
| White and Asian | 1 |
| White and Black African | 1 |
| White and Black Caribbean | 1 |
| White and Indian | 1 |
| White and Native South American | 1 |
| White Eastern European | 1 |
| White Eastern/Southern European | 1 |
| White English/Welsh/Scottish/Northern Irish/British | 72 |
| White European | 1 |
| White French | 1 |
| White Greek Cypriot | 1 |
| White Hungarian | 1 |
| White Irish | 3 |
| White Jewish | 1 |
| White Latino | 1 |
| White Other | 1 |
| White Portuguese | 1 |
| White Sardinian, Ashkenazi and Italian | 1 |
| White Slavic | 3 |
| White South African | 1 |
| White Ukrainian | 1 |
| **Total** | **117** |

**Supporting Information B**

***Table S2.*** Participants’ levels of education

| **Level of education** | **N** |
| --- | --- |
| Associate’s degree | 1 |
| Diploma | 9 |
| Master’s degree | 19 |
| PhD | 4 |
| Professional degree | 1 |
| Secondary school | 10 |
| Sixth form or college | 39 |
| Undergraduate degree | 31 |
| Other | 3 |
| **Total** | **117** |

**Supporting Information C – Psychometric properties of our experimental tasks**

*PLF Emotion Recognition Task*

In the current study, the PLF Emotion Recognition task demonstrated strong reliability. Internal consistency, measured by Cronbach’s alpha, was α = 0.86. Split-half reliability was also excellent, with a Spearman-Brown coefficient of 0.92. In previous work, our lab has examined emotion recognition performance on this task over two days (Gracey, 2025). Using these data, we have calculated test-retest reliability using intraclass correlation coefficients (ICCs) with a two-way mixed effects model and consistency definition. These analyses showed good test-retest reliability [ICCs = 0.731-0.787; Gracey, 2025] across emotion categories. Our task also demonstrates strong concurrent validity: accuracy on the point-light face emotion recognition task is strongly correlated with performance on a validated (see Chard et al., 2019; Edey et al., 2017; Schuster et al., 2022) point-light walker emotion recognition task [r = .70, p < .001; data from Schuster et al., 2022]. Finally, this task also demonstrates excellent discriminant validity, showing sensitivity to differences in emotion recognition across various groups: it effectively distinguishes autistic and non-autistic individuals [t = 2.78, p = .007, d = 0.72; Keating et al., 2022], those with and without alexithymia [t = 2.63, p = .009, d = 0.56; Keating & Cook, 2023], and people with and without Parkinson’s disease [e.g., t = 2.30, p_bonf_ = .027; Gracey, 2025) — differences that align with established research (Keating & Cook, 2020; Kinnaird, Stewart & Tchanturia, 2019; Gray & Tickle-Degnen, 2010). In sum, our PLF Emotion Recognition Task has good internal consistency, test-retest reliability, construct validity, and discriminant validity.

*Emotional Vocabulary Test*

In the current study, the Emotional Vocabulary Test had strong internal consistency and split-half reliability for total test scores [Cronbach’s alpha = 0.858; Spearman-Brown Coefficient = 0.878] and conceptual distance scores [Cronbach’s a = 0.986; Spearman-Brown Coefficient = 0.943]. Beyond this, the EVT – adapted with only minor adjustments from prior work (e.g., Nook et al., 2017; Nook et al., 2018; Nook et al., 2020) – also demonstrates good construct and concurrent validity. Emotional vocabulary scores show a significant correlation with alexithymia, as measured by validated questionnaires here [TAS; R = .182, p < .05], and with general vocabulary, as assessed by validated, standardized instruments (e.g., WASI-II) in other work [r = 0.36, p < .001; Lee, Keating, Catmur & Bird, pre-print]. These relationships indicate that the task reflects both language ability and the capacity to understand and describe emotions, supporting its validity as a measure of emotional vocabulary. Finally, this task has shown excellent discriminant validity, showing sensitivity to differences in emotional vocabulary between those with and without alexithymia here [t(115) = 2.090, = .039, d = 0.39], and distinguishing professional writers (who compose work about one’s own emotions or the emotions of fictional characters) and non-writers in other work [t = 2.84, p = .005, d = 0.49; Lee et al., pre-print]. In sum, our Emotional Vocabulary Test has good internal consistency, construct validity, and discriminant validity.

*EmoMap*

In this study, EmoMap had strong internal consistency, with excellent split-half reliability both for distance scores [distance between cluster: Spearman Brown Coefficient = 0.94; distance within cluster: Spearman-Brown Coefficient = 0.90] and emotional consistency scores [Spearman-Brown Coefficient = 0.985-0.994 across conditions]. In other work, we have assessed EmoMap performance across two sessions at least a week apart (Rybicki et al., in prep.), and then calculated test-retest reliability using intraclass correlation coefficients (ICCs). Our analyses on these data demonstrate very good test-retest reliability, both for distance between [ICC = 0.87] and within clusters [ICC = 0.82], and for our emotion-specific variables [distance between angry and happy = 0.89; distance between angry and sad = 0.81; distance between happy and sad = 0.87; distance within angry = 0.81; distance within happy = 0.71; distance within sad = 0.81]. This provides compelling evidence for gold-standard test-retest reliability, with stable and reliable measurements across repeated assessments. Moreover, our EmoMap paradigm demonstrates good concurrent/construct validity. Distances between and within clusters show a significant negative relationships with alexithymia [distance between clusters: F(1,267) = − 5.92, *p* < 0.05; distance within clusters: F(1,267) = − 6.16, *p* < 0.05; Keating & Cook, 2023] and positive relationships with interoceptive accuracy [distance between clusters: R = .217, p = .002; distance within clusters: R = .178, p = 011; Keating et al., in prep], as measured by gold-standard, validated scales (Toronto Alexithymia Scale, Interoceptive Accuracy Scale). These relationships are expected based on established research (Erbas et al., 2014; Ventura-Bort, 2021); those with high interoceptive accuracy and low alexithymia should be best able to identify and distinguish their internal emotional signals. Finally, this task has strong discriminant validity. In Keating & Cook (2023), participants reaching threshold for alexithymia (61+) had significantly smaller distance between clusters [mean(SEM) = 17.25(0.64)] than those without alexithymia (<61) [mean(SEM) = 18.85(0.24); t(269) = 2.48, p = .014, d = 0.43], which aligns with established research (Erbas et al., 2014). Similarly, this task can distinguish people high and low in interoception: participants with low interoceptive accuracy have smaller distances between clusters [mean(SEM) = 16.77(0.26)] than those with high interoceptive accuracy [mean(SEM) = 17.88(0.27); t(200) = -2.96, p = .003, d = -0.42]. In sum, our EmoMap paradigm has good internal consistency, test-retest reliability, construct validity, and discriminant validity.

**Supporting Information D – Analyses after exclusions based on AQ cut-off**

In our sample, 15 non-autistic participants scored above the AQ cut-off for potential autism (≥ 26), while six autistic participants scored below it (<26). To ensure that the absence of significant group differences was not due to high autistic traits in the non-autistic group and low traits in the autistic group – which could reduce between-group differences – we excluded these participants and repeated our analyses.

**After exclusions, still no differences between groups in emotional consistency**

First, to compare the consistency of emotional experiences across participant groups, we conducted a linear mixed effects model with emotional consistency as the dependent variable, emotion (angry, happy, sad), group (autistic, non-autistic), the interaction between emotion and group [independent variables], age, sex, non-verbal reasoning ability, years of education, alexithymia, emotional vocabulary score, between valence conceptual distance, within valence conceptual distance, and mean definition word count [control variables] as predictors, and subject number as a random intercept. As in our whole sample, we found that within valence conceptual distance was a significant positive predictor of emotional consistency [F(1,84) = 4.39, p = .039]. In line with our analyses in the whole sample, there was no main effect of group [F(1,196.34) = 1.42, p = .814], nor an emotion x group interaction [F(2,188) = 1.08, p = .342]. Our Bayesian follow-up analyses provided moderate evidence that there was no main effect of group [BF_01_ = 5.60] and no emotion x group interaction [BF_01_ = 5.62].

**After exclusions, still no differences between groups in emotion differentiation for distinct emotional states**

Next, to compare how well the autistic and non-autistic participants could differentiate distinct emotional states, we constructed a linear mixed effects model with distance between clusters as the dependent variable, emotion pair (angry-happy, angry-sad, happy-sad), group (autistic, non-autistic), the interaction between emotion pair and group [independent variables], age, sex, non-verbal reasoning, years of education, alexithymia, emotional vocabulary score, within valence conceptual distance, between valence conceptual distance, and mean definition word count [control variables] as predictors. As in our whole sample, there was a significant main effect of emotion pair [F(2,188) = 62.13, p < .0001], and between valence conceptual distance [F(1,84) = 5.58, p = .021] and non-verbal reasoning [F(1,84) = -7.86, p = .006] were significant predictors of distance between clusters. In line with what we found in our whole sample, there was no main effect of group [F(1,108.98) = 0.00, p = .953] nor an emotion x group interaction [F(2,188) = 1.28, p = .280]. Again, our Bayesian analyses provided anecdotal evidence that there was no main effect of group [BF_01_ = 1.90] and moderate evidence that there was no emotion x group [BF_01_ = 5.08] interaction.

**After exclusions, still no differences between groups in emotion differentiation for similar emotional states**

To compare how well the autistic and non-autistic participants could differentiate more similar emotional states, we constructed a linear mixed effects model with distance within clusters as the dependent variable, emotion pair (angry-happy, angry-sad, happy-sad), group (autistic, non-autistic), the interaction between emotion pair and group [independent variables], age, sex, non-verbal reasoning, years of education, alexithymia, emotional vocabulary score, within valence conceptual distance, between valence conceptual distance, and mean definition word count [control variables] as predictors. As in our whole sample, there was a significant main effect of emotion [F(2,188) = 12.52, p < .0001], and between valence conceptual distance [F(1,84) = 4.43, p = .038] and non-verbal reasoning [F(1,84) = -17.38, p < .0001] were significant predictors of distance between clusters. In line with what we found in rhe whole sample, there was no main effect of group [F(1,107.83) = 0.45, p = .502] nor an emotion x group interaction [F(2,188) = 1.47, p = .232]. Again, our Bayesian analyses provided anecdotal evidence that there was no main effect of group [BF_01_ = 2.40] and moderate evidence that there was no emotion x group [BF_01_ = 4.25] interaction.

**After exclusions, still no differences between groups in the understanding of emotion concepts**

Next, we compared the emotional vocabulary test scores of the autistic and non-autistic participants. To do so we ran a non-parametric multiple regression of emotional vocabulary as a function of group (autistic, non-autistic), age, sex, non-verbal reasoning, years of education, alexithymia, and mean definition word count [control variables]. As in our whole sample, mean definition word count [t(87)= 3.61, p<.001] was a positive predictor, and age [t(87)= -3.45, p < .001] was a negative predictor of emotional vocabulary score. In line with what we found in our whole sample, there were no significant differences between the autistic participants and non-autistic participants in emotional vocabulary score [t(87)=-0.25, p = .800]. A follow-up Bayesian independent sample t-test revealed moderate evidence for this null effect [BF_01_ = 3.69].

**After exclusions, still no differences between groups in the overlap of semantic conceptions of emotion**

Following this, to determine whether autistic or non-autistic people have more differentiated conceptions of emotions with the same and opposite valences, we constructed two simple linear models as a function of group (autistic, non-autistic), age, sex, non-verbal reasoning, years of education, alexithymia, and mean definition word count [control variables]. In line with what we found in our whole sample, across both models, mean definition word count was a negative predictor [between valence: F(1,87)= -8.96, p = .004; within valence: F(1,87) = -10.12, p= .002] and age [between valence: F(1,87)= 6.04, p =.016; within valence: F(1,87)= 11.77, p < .001] was a positive predictor: those who provided longer definitions, and those younger in age, tended to have lower conceptual distance scores, both for same-valence and opposite-valence emotions. As in our whole sample, there was no effect of group [between valence conceptual distance: F(1,87) = 0.61, p = .436; within valence conceptual distance: F(1,87) = 1.51, p= .223] in both models. As previously, follow-up Bayesian independent sample t-tests provided moderate evidence that there was a null effect of group for both between valence conceptual distance [BF_01_= 4.42] and within valence conceptual distance [BF_01_= 3.21].

**Conclusion**

In sum, after excluding autistic participants with low AQ scores (<26) and non-autistic participants with high AQ scores (26+), our results remained the same: there were no differences between the autistic and non-autistic participants in emotional consistency, emotion differentiation for distinct emotional states, emotion differentiation for similar emotional states, the understanding of emotion concepts, the differentiation of same-valence emotion concepts, and the differentiation of opposite-valence emotion concepts.

**Supporting Information E – Investigating whether there are differences between the autistic and non-autistic participants on our variables of interest when simpler statistical models are used**

To rule out the possibility that the lack of significant group differences was due to the complexity of our statistical models, we conducted additional analyses using simpler models with fewer predictors. Crucially, even with these simplified models, we still found no significant differences between autistic and non-autistic participants in emotional consistency, differentiation of distinct and similar emotional states, understanding of emotion concepts, or differentiation of same-valence and opposite-valence emotion concepts (see below).

**No differences between groups in emotional consistency**

First, to compare the consistency of emotional experiences across participant groups, we conducted a linear mixed effects model with emotional consistency as the dependent variable, emotion (angry, happy, sad), group (autistic, non-autistic), the interaction between emotion and group and alexithymia as predictors, and subject number as a random intercept. There was no main effect of group [F(1,307.58) = 0.04, p = .844] and no emotion x group interaction [F(2,230) = 0.43, p = .649], suggesting there were no differences in emotional consistency between the autistic and non-autistic participants across all three emotions.

**No differences between groups in emotion differentiation for distinct emotional states**

To test whether autistic adults have less differentiated experiences of distinct emotions than non-autistic adults, we constructed a linear mixed effects model with distance between clusters as the dependent variable, emotion pair (angry-happy, angry-sad, happy-sad), group (autistic, non-autistic), the interaction between emotion pair and group, and alexithymia as predictors, and subject number as a random intercept. There was no main effect of group [F(1,158.99) = 2.42, p = .122] nor an interaction between emotion pair and group [F(2,230) = 1.68, p = .189], suggesting that there were no differences between autistic and non-autistic participants in the differentiation of distinct emotional states.

**No differences between groups in emotion differentiation for similar emotional states**

To test whether autistic adults have less differentiated experiences of similar emotions than non-autistic adults, we constructed a linear mixed effects model with distance within clusters as the dependent variable, emotion (angry, happy, sad), group (autistic, non-autistic), the interaction between emotion and group, and alexithymia as predictors, and subject number as a random intercept. There was no main effect of group [F(1,157.24) = 0.17, p = .679] nor an interaction between emotion pair and group [F(2,230) = 1.12, p = .327], suggesting that there were no differences between autistic and non-autistic participants in the differentiation of distinct emotional states.

**No differences between groups in levels of understanding of emotion concepts**

To assess the understanding of emotion concepts we compared the emotional vocabulary test scores of the autistic and non-autistic participants. To do so we ran a non-parametric multiple regression of emotional vocabulary as a function of group (autistic, non-autistic) and alexithymia. There were no significant differences between the autistic participants and non-autistic participants in emotional vocabulary score [t(114) = -0.46, p = .644].

**No differences between groups in the differentiation of semantic emotion concepts**

Following this, to determine whether autistic or non-autistic people have more differentiated conceptions of emotions with the same and opposite valences, we constructed two simple linear models as a function of group (autistic, non-autistic) and alexithymia. Across both models, there were no differences between groups in the differentiation of semantic emotion concepts between autistic and non-autistic participants [between valence: F(1,114) = 0.01, p = .942; within valence: F(1,114) = 0.46, p = .499].

**Conclusion**

In sum, even with these simplified models, we still found no significant differences between autistic and non-autistic participants in emotional consistency, differentiation of distinct and similar emotional states, understanding of emotion concepts, or differentiation of same-valence and opposite-valence emotion concepts (see below).

**Supporting Information F – Examining whether there were any interactions with sex in our primary analyses.**

Here, aimed to test whether there were significant sex by group interactions in our analyses comparing the autistic and non-autistic participants on emotion-processing. To fulfil this aim, we added a sex x group interaction to our linear mixed models of emotional consistency, distance between clusters, distance within clusters, emotional vocabulary, between valence conceptual distance and within valence conceptual distance. If we found a significant sex by group interaction in these analyses, this would indicate either that (1) there was a significant difference between autistic and non-autistic individuals in one sex but not another (e.g., males but not females), or (2) that there was a significant difference between males and females in one diagnostic group but not another (e.g., autistic but not non-autistic). Nevertheless, in these analyses, there was no significant sex x group interaction for emotional consistency, distance between clusters, distance within clusters, emotional vocabulary score, between valence conceptual distance and within valence conceptual distance [all p >.05]. As such, there were no differences between the autistic and non-autistic participants in our emotion-related variables, irrespective of sex.

**References**

Chard, J., Edey, R., Yon, D., Murphy, J., Bird, G., & Press, C. (2019). Atypical emotion recognition from bodies is associated with perceptual difficulties in healthy aging. *Journal of Experimental Psychology: Human Perception and Performance*, *45*(6), 803.

Edey, R., Yon, D., Cook, J., Dumontheil, I., & Press, C. (2017). Our own action kinematics predict the perceived affective states of others. *Journal of Experimental Psychology: Human Perception and Performance*, *43*(7), 1263.

Erbas, Y., Ceulemans, E., Lee Pe, M., Koval, P., & Kuppens, P. (2014). Negative emotion differentiation: Its personality and well-being correlates and a comparison of different assessment methods. *Cognition and emotion*, *28*(7), 1196-1213.

**Gracey, M.** (2025). Exploring emotion recognition in people with Parkinson’s (Doctoral dissertation, University of Birmingham). University of Birmingham eTheses Repository.

Gray, H. M., & Tickle-Degnen, L. (2010). A meta-analysis of performance on emotion recognition tasks in Parkinson’s disease. *Neuropsychology*, *24*(2), 176.

Keating, C. T., & Cook, J. L. (2020). Facial Expression Production and Recognition in Autism Spectrum Disorders: A Shifting Landscape. *Child and adolescent psychiatric clinics of North America*, *29*(3), 557-571.

Keating, C. T., & Cook, J. L. (2023). The inside out model of emotion recognition: how the shape of one’s internal emotional landscape influences the recognition of others’ emotions. *Scientific Reports*, *13*(1), 21490.

Keating, C. T., & Cook, J. L. (in prep.). Emotion differentiation as a protective factor for mental health difficulties.

Keating, C. T., Fraser, D. S., Sowden, S., & Cook, J. L. (2022). Differences between autistic and non-autistic adults in the recognition of anger from facial motion remain after controlling for alexithymia. *Journal of autism and developmental disorders*, *52*(4), 1855-1871.

Kinnaird, E., Stewart, C., & Tchanturia, K. (2019). Investigating alexithymia in autism: A systematic review and meta-analysis. *European Psychiatry*, *55*, 80-89.

Lee, K. S., Keating, C. T., Catmur, C. & Bird, G. (pre-print) Language talents and emotional self-awareness: Low alexithymic traits in writers are related to interoceptive accuracy but not emotion vocabulary expertise.

Nook, E. C., Sasse, S. F., Lambert, H. K., McLaughlin, K. A., & Somerville, L. H. (2017). Increasing verbal knowledge mediates development of multidimensional emotion representations. *Nature human behaviour*, *1*(12), 881-889.

Nook, E. C., Sasse, S. F., Lambert, H. K., McLaughlin, K. A., & Somerville, L. H. (2018). The nonlinear development of emotion differentiation: Granular emotional experience is low in adolescence. *Psychological science*, *29*(8), 1346-1357.

Nook, E. C., Stavish, C. M., Sasse, S. F., Lambert, H. K., Mair, P., McLaughlin, K. A., & Somerville, L. H. (2020). Charting the development of emotion comprehension and abstraction from childhood to adulthood using observer-rated and linguistic measures. Emotion, 20(5), 773–792.

Rybicki, A., Keating, C. T., Cook, J. L. & Hickey, C. (in prep.). Dopaminergic modulation of emotion differentiation.

Schuster, B. A., Sowden, S., Rybicki, A. J., Fraser, D. S., Press, C., Holland, P., & Cook, J. L. (2022). Dopaminergic modulation of dynamic emotion perception. *Journal of Neuroscience*, *42*(21), 4394-4400.

Ventura-Bort, C., Wendt, J., & Weymar, M. (2021). The role of interoceptive sensibility and emotional conceptualization for the experience of emotions. *Frontiers in psychology*, *12*, 712418.
